# Decreased Expression of MPC2 Contributes to Aerobic Glycolysis and Colorectal Cancer Proliferation by Activating mTOR Pathway

**DOI:** 10.1155/2021/6618837

**Published:** 2021-03-15

**Authors:** Manzila Kuerbanjiang, Lei Gu, Chunjie Xu, Wen Ting Xu, Siyuan Wen, Hanbing Xue, Qing Xu

**Affiliations:** ^1^Department of Gastrointestinal Surgery, Renji Hospital, Shanghai Jiao Tong University School of Medicine, Shanghai, China; ^2^China Welfare Association International Peace Maternal and Child Health Care Center, Shanghai, China; ^3^Department of Gastroenterology and Hepatology, Key Laboratory of Gastroenterology and Hepatology, Ministry of Health, Renji Hospital, Shanghai Jiao Tong University School of Medicine, Shanghai Institute of Digestive Disease, Shanghai, China

## Abstract

Mitochondrial Pyruvate Carrier 1 (MPC1), one of the rate-limiting proteins involved in glycolysis metabolism, has been demonstrated as a tumor inhibitor in several cancers. This study was conducted with the aim of exploring the role and underlying mechanisms of MPC2 in colorectal cancer (CRC). Here, we found that MPC2 expression was decreased in CRC samples. According to the analysis on our TMA data, lower expression of MPC2 is correlated with a higher incidence of distant metastasis and lymph node invasion, bigger tumor size, low survival rate of patients, and advanced T stages. Functionally, in vivo/vitro experiments showed that MPC2 knockdown induced CRC cell proliferation and growth, while MPC2 overexpression inhibited the proliferation and growth of CRC. Further study demonstrated that MPC2 knockdown resulted in aerobic glycolysis in CRC cells. Similarly, MPC2 overexpression in CRC cells also caused inhibited aerobic glycolysis. Further study found that MPC2 knockdown in CRC cell lines activated the mTOR signaling pathway, and the addition of rapamycin reversed the promoting effect of MPC2 knockdown on CRC proliferation and glycolysis. Likewise, the addition of MHY1485 also reversed the MPC2 overexpression's role in hindering aerobic glycolysis in CRC cells. Collectively, our study established that low expression of MPC2 led to CRC growth as well as aerobic glycolysis through the regulation of the mTOR pathway in CRC cells, indicating a potential biomarker and therapy target for CRC.

## 1. Introduction

Colorectal Carcinoma (CRC), as a prevailing type of cancer worldwide, ranked the third-highest cancer-related death factor in the United States [1, 2]. The prognosis of CRC patient stage at diagnosis and 5-year survival rate of CRC patients at stage 1 and stage 2 could reach 70%, while less than 10% for stage IV [3]. Therefore, prognostic biomarkers as well as therapy target are urgent to be identified to improve CRC patients' survival.

Mitochondrial Pyruvate Carrier (MPC) is a heteroligomeric complex containing two members, MPC1 and MPC2 [4–7]. The complex localizes in the integral inner mitochondrial membrane and is in charge of conducting pyruvate from the cytosol into the mitochondria, one of the rate-liming steps in glycolysis, and links glycolysis with mitochondrial oxidative phosphorylation (OXPHOS) [5, 8]. An increasing number of studies showed downregulated expression of the MPC complex in different cancers, such as prostate cancer and hepatocellular carcinoma, and restoring MPC2 in human tumor cells inhibited melanoma cell proliferation and migration [9–11]. Moreover, a study showed that MPC2 predicted poor survival in patients with isocitrate dehydrogenase-mutant glioma [12], although the potential role of MPC2 in cancer, the clinical relevance, and molecular mechanism underlying its influence on CRC progression remain unknown.

Metabolic reprogramming has been considered one of the hallmarks of cancer, with the Warburg effect being a common feature [13, 14]. Warburg effect is featured as high glucose uptake and utilization to provide cancer with increased bioenergetics and biosynthesis in anorexic and hypoxic conditions [8]. Therefore, the association and potential regulatory mechanism of glycolysis in cancer are to be studied.

In this study, we showed low expression of MPC2 CRC samples, which resulted in aerobic glycolysis via activating the mTOR pathway and promoted CRC proliferation.

## 2. Materials and Methods

### 2.1. Database, Patients, and Clinical Tissue Specimens

We analyzed the MPC2 mRNA expression in 327 samples (including 286 CRC tissues and 41 tissues adjacent to cancer) from The Cancer Genome Atlas (TCGA) database and 17 matched samples from GEO [15]. OncoLnc (http://www.oncolnc.org) was used to link survival data of patients in the TCGA database to normalized MPC2 mRNA; patients were divided into the high group (top thirty percentile) and the low group (lower thirty percentile). The Kaplan plot curves and the log-rank *p* value were analyzed automatically by OncoLnc. The tissue microarray chip consists of three hundred ninety-two CRC pairs of adjacent noncancerous and tumor samples from patients diagnosed with CRC in Renji Hospital, China. The ethics committee of Renji Hospital approved the study, and every patient was assigned informed consent.

### 2.2. Cell Culture

LOVO, HCT116, RKO, and SW620 as human colon carcinoma cell lines were provided by ATCC. Dulbecco's modified Eagle's medium (DMEM, Hyclone) with 10% fetal bovine serum (FBS, Gibco) as well as 1% penicillin and 1% streptomycin was used to culture the cells in an incubator at 5% CO_2_ and 37°C.

### 2.3. Lentivirus Transfection

Lentiviral vectors for the short-hairpin RNA (shRNA) for MPC2 were generated by GenePharma (Shanghai, China). We transferred the shRNA sequence for MPC2 into CRC cell lines and generated shRNA-MPC2#1 and shRNA-MPC2#2 with 10 mg/mL polybrene (Sigma, H9268). Moreover, the negative control was set as shNC-MPC2. The expression of MPC2 (MPC2-OE) was generated by transfecting full-length human MPC2 cDNA, where the negative control (vector) was lentivirus-NC. Supplementary Table S1 lists the sequences. Transfection of shRNA was performed according to the manufacturer's protocol.

### 2.4. RNA Extraction and RT-qPCR

Total RNA was extracted from cell samples by the Simply P Total RNA Extraction Kit (BSC51S1, Bioer Technology, China). Reverse transcription to cDNA was conducted by the PrimeScript RT reagent Kit (RR037Q, Takara Biotech, China). The target gene's relative expression to 18S RNA was calculated by the *ΔΔ*Ct method. Supplementary Table S1 lists all primers designed for the RT-qPCR in this study.

### 2.5. Protein Extraction and Western Blotting

The RIPA buffer (Sangon Biotech, China), as well as 1% inhibitors of phosphatase and protease (Sangon Biotech, China), was applied to extract total protein. A BCA Protein Assay Kit (A53225, Thermo Fisher) was selected for the measurement of protein concentration. Western blotting was performed as previously described. Such primary antibodies as MPC2 (1 : 1000, ab236584, Abcam, Cambridge, MA), *β*-actin (1 : 1000, 20536-1-AP, Proteintech, China), mTOR (1 : 1000, 2983, CST, USA), and p-mTOR (1 : 1,000, 2971, CST, USA) were adopted. Secondary antibodies were HRP-conjugated goat anti-rabbit IgG provided by Proteintech, China (1 : 5000, SA00001-2).

### 2.6. Immunohistochemistry (IHC)

IHC staining was conducted following a standard procedure as previously described [16]. Tissues were embedded with paraffin and incised into 4 *μ*m thick sections. Dewaxing the sections by xylene and hydrating by alcohol were then performed, followed by antigen retrieval via sodium citrate (Sangon Biotech, China) and endogenous peroxidase blocking by 0.3% H_2_O_2_. Bovine serum albumin (BSA, Sangon Biotech) was used to block nonspecific sites, and MPC2 (1 : 100, ab236584, Abcam) and Ki67 (1 : 100, 27309-1-AP, Proteintech, China) were used to incubate the slices for 8 hrs then with secondary antibody (1 : 100, SA00001-2, Proteintech, China) for 60 minutes. The DAB Substrate kit (# 8059S, CST) was used for visualization; then, nuclei were stained with hematoxylin. Further dehydration was performed with alcohol and sealing with neutral resin. IHC stainings were qualified using a grade scoring method, and it was scored from 1 to 4 as no, weak, moderate, and strong staining, respectively [17, 18]. Besides, samples were divided into two groups according to their MPC2 staining: the low level group (scores 1 and 2) and the high level group (scores 2 and 3).

### 2.7. Cell Growth and Colony Formation Assays

The cell viability was measured by the Cell Counting Kit-8 (CK04, Dojindo, Japan) using the method as previously described [19]. Cells were inoculated with suspension of 100 *μ*l/well (3000 cells/well) in a 96-well plate and cultured in an incubator (at 37°C, 5% CO_2_) for 24, 48, 72, and 96 h after the treatment. After being added with CCK-8 solution (10 *μ*l/well), the cells were incubated for 2 h at 37°C. Then, the absorbance at 450 nm was measured in a microplate reader.

For the clonogenic assay, we used 6-well plates (3000 cells/well) to inoculate cells. After two weeks, fixation by 4% paraformaldehyde and staining by 0.5% crystal violet for 30 min were performed.

### 2.8. Extracellular Acidification and Oxygen Consumption Rate Assays

Regarding the cultured cells, the assays for the oxygen consumption rate (OCR) and extracellular acidification rate (ECAR) were detected as previously reported under the support of the Seahorse XF96 Flux Analyzer (Seahorse Bioscience, Agilent) following the protocol of the manufacturer [20]. The Seahorse XF Cell Mito Stress Test Kit and Seahorse XF Glycolysis Stress Test Kit (103010-100 and 103020-100, Agilent Technologies, USA) were used to examine OCR and ECAR, respectively. To be specific, we inoculated cells in an XF 96-well plate (1 × 10^4^/well) with the indicated treatment. After baseline measurements, for the glycolytic stress test, 10 mmol/L glucose, 1 mmol/L oligomycin (oxidative phosphorylation inhibitor), and 50 mmol/L 2-deoxyglucose (2-DG, oxidative phosphorylation inhibitor) were applied to every well in order at a given time. In the mitochondrial stress test, 1 mmol/L oligomycin, 1 mmol/L FCCP (reversible inhibitor of oxidative phosphorylation), 0.5 mmol/L rotenone (Rote, the mitochondrial complex I inhibitor), and 0.5 mmol/L actinomycin A (AA, mitochondrial complex III inhibitor) were sequentially added to the wells. Seahorse XF-96 Wave software was adopted for data analysis. Glycolytic flux and maximal respiratory capacity were measured as previously reported [21, 22]. An increase in ECAR after the glucose treatment indicated glycolytic flux; total elevation in OCR above basal respiration after FCCP treatment indicated maximal respiratory capacity. OCR and ECAR were measured in pmols/minute and mpH/minute, respectively.

### 2.9. Tumor Orthotopic Xenograft Model

The orthotopic xenograft model for CRC was generated following the method previously reported [23]. We used 0.5% pentobarbital to anesthetize nude mice. The abdominal cavity was exposed through a midline laparotomy. 1 × 10^6^ luciferase-expressing HCT116 or HT29 cell suspension was injected directly into the submucosa of the ileocecum. The growth of the tumor was monitored by the IVIS spectrum (Caliper Life Science, USA) intraperitoneal injection of D-luciferin (150 mg; Promega, catalog) and gas anesthesia. Living Image software (version 4.5.3) was used to quantify the luciferin emission. Four weeks later, we sacrificed mice, the xenograft tumor of which was separated, weighed, and fixed with 4% paraformaldehyde. The International Standards of Animal Welfare were followed during the whole animal studies, which were approved by the Research Ethics Committee of Renji Hospital.

### 2.10. Statistical Analysis

We repeated all in vitro experiments no less than three times. GraphPad Prism (San Diego, CA) was applied to the overall statistical analyses. To figure out how categorical clinical variables in CRC patients correlated with MPC2 expression, Student's *t*-test or chi-squared analysis was applied to calculate the measurement data such as age and tumor size or to calculate categorical variables including sex and the presence of patients' distant metastasis and lymph node invasion and to rank data like T stages. The two-way ordered categorical data were calculated by Spearman's rank correlation. The data generated by patient survival curves were analyzed by the log-rank test as well as the Kaplan-Meier method. The mean ± SD was adopted to express the measurement data. The significant level in this study is 5%. The significant levels are the following: ^∗^*p* value < 0.05, ^∗∗^*p* value < 0.01, ^∗∗∗^*p* value < 0.001, and ^∗∗∗∗^*p* value < 0.0001.

## 3. Results

### 3.1. MPC2 Was Downregulated in CRC Tissue and Correlated with Poor Prognosis of CRC

**Figure 1:**
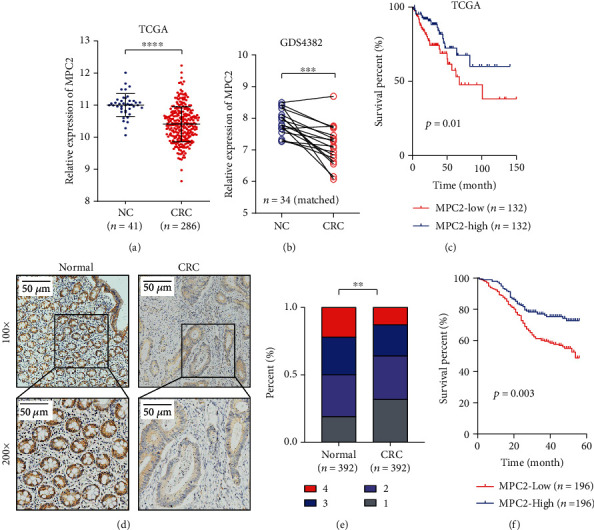
MPC2 is downregulated in tumor tissue and correlated with a poor prognosis of Colorectal Carcinoma (CRC). **(**a) The mRNA expression of MPC2 in the CRC tissues compared to tissue adjacent to cancer in The Cancer Genome Atlas (TCGA) database. (b) The mRNA expression of MPC2 in the CRC tissues compared to paired tissue adjacent to cancer selected from the GDS4382 database. (c) Overall survival analyzed by a log-rank test and the Kaplan-Meier method according to the MPC2 mRNA expression from the TCGA dataset. (d) Representative IHC images for MPC2 expression of normal tissues and matched CRC tissues from TMA. (e) IHC score of MPC2 expression of normal tissues and matched CRC tissues from TMA. (f) Overall survival analyzed through the log-rank test and Kaplan-Meier method based on the MPC2 mRNA expression from TMA. ^∗^*p* < 0.05, ^∗∗^*p* < 0.01, ^∗∗∗^*p* < 0.001, and ^∗∗∗∗^*p* < 0.0001.

According to the dataset from TCGA and the GEO, compared with paired tissue adjacent to cancer, the mRNA expression of MPC2 decreased in CRC tissues (Figures 1(a) and 1(b)). Notably, Kaplan-Meier survival analysis indicated shorter overall survival for patients that had a lower level of MPC2 expression (*p* = 0.01) (Figure 1(c)). IHC staining in a TMA containing 392 CRC specimens and paired corresponding tissue adjacent to cancer was performed to validate the protein level of MPC2. The expressions were scored according to the intensity. The result of TMA analysis also showed substantially lower MPC2's protein expression in CRC tissues than those in tissues adjacent to cancer, and CRC tissues manifested a lower staining score (Figures 1(d) and 1(e)). Therefore, our data showed downregulation of MPC2 expression in CRC tissues as compared to adjacent tissues. The association between MPC2 expression and patients' clinicopathological characteristics was analyzed to explore the clinical significance of MPC2.  Table 1 implies the negative correlation of tumor size, incidence of distant metastasis, and lymph node invasion, as well as T staging, with the expression of MPC2. Besides, the survival of patients with lower MPC2 expression was lower than that of patients with higher MPC2 expression levels, as demonstrated by Kaplan-Meier survival analysis (Figure 1(f)).

### 3.2. MPC2 Suppressed Proliferation of CRC Cells *In Vitro* and *In Vivo*

**Figure 2:**
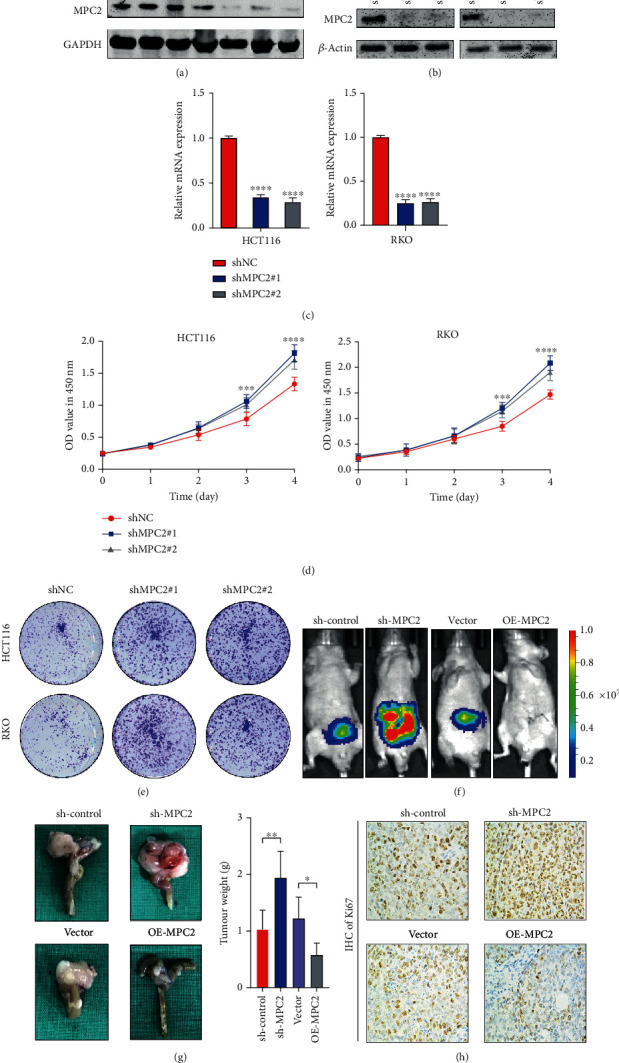
MPC2 suppressed CRC growth in vitro and *in vivo*. (a) The protein expression of MPC2 in seven CRC cell lines was detected using western blotting. (b) The inhibition efficiency in RKO cells (right) and HCT116 cells (left) using western blotting. (c) The inhibition efficiency in HCT116 (left) and RKO (right) cells showed by qRT-PCR (relative to shNC). (d) The viability of HCT116 (left) as well as RKO cell (right) transfected with shMPC2 or shNC detected with CCK-8 assay. (e) Colony formation ability of HCT116 and RKO cells through transfection by shNC or sh-MPC2. (f) Representative bioluminescence images of mice orthotopically injected with mcherry and luciferase-expressing HCT116 cells transfected with shNC and shMPC2 as well as vector and OE-MPC2. (g) The representative images in gross and weight of tumor mass from orthotopic mouse model among shNC, sh-MPC2, vector, and OE-MPC2 groups (Student's *t*-test). (h) Ki67's expression in the orthotopic tumor tissues (shNC, sh-MPC2, vector, and OE-MPC2, *n* = 5). ^∗^*p* < 0.05, ^∗∗^*p* < 0.01, ^∗∗∗^*p* < 0.001, and ^∗∗∗∗^*p* < 0.0001.

Next, we measured the expression of protein MPC2 and mRNA in several CRC cell lines (Figure 2(a), Supplementary Figure S1A). HCT116 and RKO cells were selected for investigating the effect of MPC2 knockdown because of their relatively higher expressions, while HT29 and SW620 cells were selected for investigating the effect of MPC2 overexpression on CRC cells according to the lower expressions. Both mRNA and protein expressions of MPC2 were markedly downregulated after transfecting with shRNA#1 (shMPC2#1) and shRNA#2 (shMPC2#2) in HCT116 and RKO cells and upregulated after transfecting with MPC2-OE in HT29 and SW620 cells (Figures 2(b) and 2(c)), Supplementary Figure S1B). Besides, as MPC is a complex, we further detected the expression of MPC1 after MPC2 knockdown or overexpression. The result showed neither mRNA level nor protein level of MPC1 altered in CRC cells with MPC2 knockdown or overexpression (Supplementary Figures S1C and S1D).

Then, we assessed the proliferation of CRC cells after MPC2 knockdown or overexpression by the CCK8 assay. It was found that knockdown of MPC2 increased proliferation of RKO and HCT116 cells compared with the control (shNC) group (Figure 2(d)), while overexpression of MPC2 decreased cell proliferation in HT29 and SW620 cells compared with the control (Supplementary Figure S1G). Further, colony formation assays showed more colonies in the shMPC2#1 and shMPC#2 groups (Figure 2(e)). These data suggested that MPC2 impaired CRC cell proliferation in vitro.

The role of MPC2 in CRC growth was further assessed by an orthotopic tumor model. The sh-MPC2 (using shRNA#2) group displayed distinctly higher tumor weight than the control group, while that of the MPC2-OE group was lower compared to the vector group (Figures 2(f) and 2(g)). Further, IHC assays of Ki67 in an orthotopic tumor revealed that knockdown of MPC2 promoted Ki67 expression while overexpression of MPC2 inhibited the expression (Figure 2(h)). These data collectively suggested the role of MPC2 in suppressing CRC growth both *in vitro* and *in vivo*.

### 3.3. Downregulated MPC2 Expression Enhanced Glycolysis in CRC

**Figure 3:**
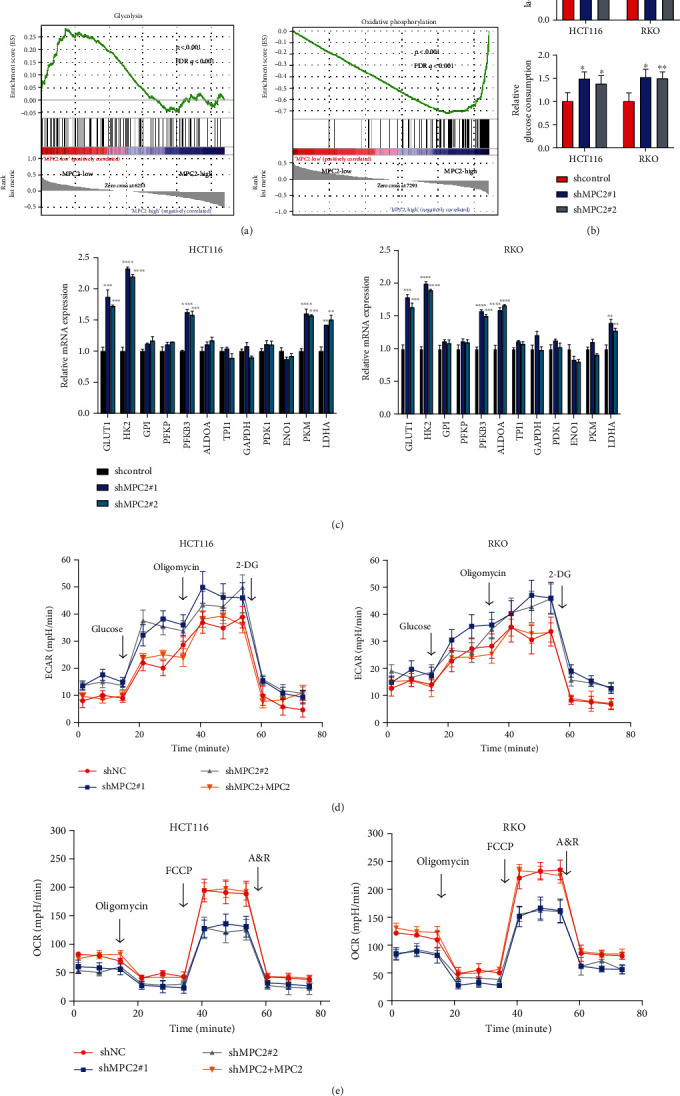
MPC2 expression suppressed glycolysis in CRC cells. (a) GSEA of MPC2 expression in CRC samples from the TCGA database. (b) Lactate production and glucose intake of CRC cells transfected with shNC or shMPC2 (relative to shNC). (c) Relative mRNA levels of glycolysis-related genes of HCT116 (left) as well as RKO cells (right) transfected with shNC or shMPC2 (relative to shNC). (d) Glycolytic function of HCT116 as well as RKO cells under the indicated treatments as measured by extracellular acidification rate (ECAR). 2-DG: 2-deoxyglucose (oxidative phosphorylation inhibitor). (e) Mitochondrial stress test of RKO cells (right) and HCT116 cells (left) and in MPC2 knockdown and/or overexpression under the indicated treatments as measured by oxygen consumption rate (OCR). A&R: actinomycin A (mitochondrial complex III inhibitor) and rotenone (the mitochondrial complex I inhibitor). ^∗^*p* < 0.05, ^∗∗^*p* < 0.01, ^∗∗∗^*p* < 0.001, and ^∗∗∗∗^*p* < 0.0001.

We performed GSEA for examining the mechanism behind downregulated MPC2 promoting CRC growth. We divided the samples from the TCGA database into the MPC-high group and the MPC2-low group based on the MPC2 expression. The results showed a negative association of MPC2 expression with glycolysis and a positive association with oxidative phosphorylation (Figure 3(a)). Therefore, glucose uptake and lactate production were detected to confirm whether knockdown and overexpression of MPC2 affect glycolysis in CRC cells. The knockdown of MPC2 promoted glucose uptake and lactate production, while overexpression of MPC2 impaired glucose intake and lactate production, as demonstrated in Figure 3(b) and Supplementary Figures S3A and S3B. In addition, glycolysis-related genes were examined, and the results showed that *GLUT1*, *HK2*, *PFKFB3*, and *LDHA* were upregulated and inhibited by MPC2 knockdown and overexpression, respectively (Figure 3(c) and Supplementary Figure S3C). Moreover, an indicator of overall glycolytic flux and extracellular acidification rate (ECAR), as well as oxygen consumption rate (OCR) indicators of mitochondrial respiration, was detected. The results indicated that MPC2 knockdown in CRC cells increased glycolytic flux and decreased maximal respiratory capacity, which was recovered by the rescue of MPC2 overexpression (Figures 3(d) and 3(e) and Supplementary Figure S2A). In contrast, MPC2 overexpression decreased glycolytic flux and increased maximal respiratory capacity (Supplementary Figures S3D and S3E). Taken together, these data suggested that MPC2 expression impaired glycolysis in CRC cells.

### 3.4. MPC2 Reduced Glycolysis Level via mTOR Pathway in CRC Cells

**Figure 4:**
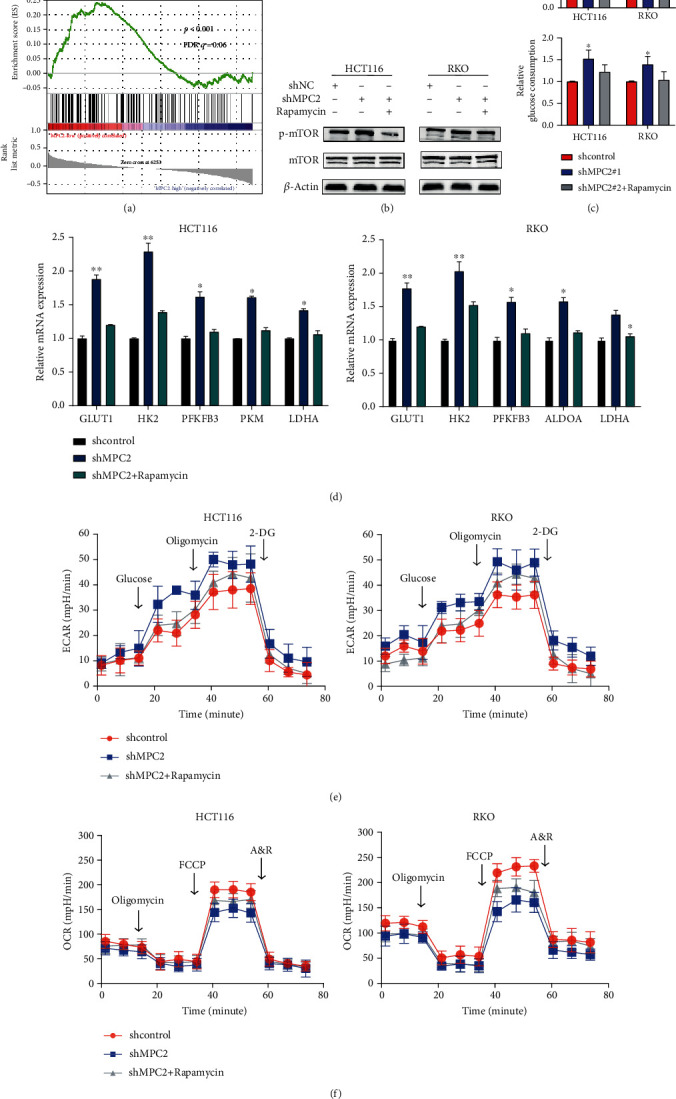
MPC2 reduced glycolysis level via mTOR pathway in CRC cells. (a) GSEA from CRC tissues from the TCGA database. (b) Western blotting images of phospho-mTOR (p-mTOR) and mTOR in HCT116 and RKO cells with shNC or shMPC2 transfection and/or rapamycin treatment (50 nmol/L). (c) Lactate production and glucose consumption of CRC cells with shNC or shMPC2 transfection and/or rapamycin. (d) Relative mRNA expression of glycolysis-related genes of HCT116 and RKO cells with shNC or shMPC2 transfection and/or rapamycin treatment. (e) Glycolytic function of RKO and HCT116 cells under the indicated treatments as measured by ECAR. (f) Mitochondrial stress test RKO cells (right) and HCT116 cells (left) under the indicated treatments as measured by oxygen consumption rate (OCR). ^∗^*p* < 0.05, ^∗∗^*p* < 0.01.

According to the result of the GSEA mentioned above, MPC2 expression was found to negatively correlate with the mTOR pathway activity (Figure 4(a)). Additionally, the mTOR signaling pathway plays a key part in regulating cell proliferation as well as glycolysis [24, 25]. Therefore, further study on the mTOR pathway was undertaken. Western blot showed that MPC2 knockdown exhibited a higher p-mTOR expression compared to the control group, while MPC2 overexpression showed a lower p-mTOR expression (Figure 4(b) and Supplementary Figure S4A). In addition, the activation of the mTOR pathway induced by MPC2 knockdown was reversed by the addition of rapamycin, an inhibitor of the mTOR pathway, while the decrease of the p-mTOR level caused by overexpressing MPC2 was reversed by the addition of mTOR activator MHY1485 (Figure 4(b) and Supplementary Figure S4A). The result showed that the effects of silencing MPC2 by shRNA#2 on glucose uptake, lactate production, glycolysis-related gene expression, ECAR, and OCR were significantly abolished by the mTOR inhibitor (Figures 4(c)–4(f)), Supplementary Figure S4B-C). Taken together, these findings indicated that MPC2 reduced glycolysis through the mTOR pathway.

## 4. Discussion

In this study, we found that downregulated MPC2 promotes CRC growth by enhancing glycolysis via the mTOR pathway. In specific, we confirmed that MPC2 expression is downexpressed in CRC tissue from an online database and TMA of our hospital, and it is negatively correlated with aggressive clinical characteristics of patients. In addition, our study demonstrated that inhibiting MPC2 promoted CRC proliferation using *in vitro* and *in vivo* models. Mechanistically, we found that downregulated MPC2 promoted glycolysis via the mTOR pathway.

A recent study found that the MPC2 mRNA level predicted a worsened survival in patients with isocitrate dehydrogenase-mutant glioma using an online database [12]. In addition, another research reported that the MPC2 level was lower in prostate cancer cells and tissue, and MPC2 predicted a better OS of patients with prostate cancer [9]. In our study, we showed the first time that MPC2 was lower in CRC tissue, and downregulated MPC2 predicted a worse OS for CRC patients using a larger amount of paired patients' tissue.

Metabolism relies on glycolysis and mitochondrial OXPHOS to produce ATP and provides energy in normal conditions. In proliferating cancer cells, aerobic glycolysis is the main process to produce ATP and supports biosynthesis even if there is enough oxygen, which is called Warburg effect [26]. This kind of metabolic reprogramming advantages cancer cell proliferation by providing energy quicker, which is recognized as a common feature of cancer, including CRC [27]. Moreover, most key molecules related to glycolysis are involved in tumor progression [28]. A glycolysis gene expression analysis of CRC showed increased glucose intake along with aerobic glycolysis in CRC tissue, and a high glycolytic character was correlated with a poorer prognosis in CRC patients [29]. In line with these previous studies, our data demonstrated that downexpression of MPC2 contributed to aerobic glycolysis in CRC cells.

The PI3K/Akt/mTOR pathway has been demonstrated as one of the central regulators of glycolysis and cancer proliferation [24, 25]. Regarding the potential mechanisms of glycolysis in CRC cells, an increasing number of studies found that activation of the mTOR pathway directly correlates with increased glycolysis in tumor cells compared with normal cells [30, 31]. A recent study demonstrated that activated Purinergic Receptor P2Y2 (P2RY2) contributed to the activation of the PI3K-mTOR pathway thus enhancing cancer glycolysis in pancreatic ductal adenocarcinoma [32]. In addition, in our study, GSEA also indicated an association between MPC2 expression and mTOR pathway. Further exploration showed that the inhibitor of the mTOR pathway could attenuate the glycolysis induced by silencing of MPC2, indicating that mTOR may participate in the role of MPC2 in glycolysis. Therefore, MPC2 may participate in glycolysis via the mTOR pathway in CRC cells. Our study showed correlation of MPC2 and mTOR pathway for the first time. A recent report revealed that extracellular lactate, the product of pyruvate, activated the Akt-mTOR signaling pathway to promote cell proliferation and prevent cell death. In addition, lactate could fuel the TCA cycle for amino acid synthesis, resulting in mTORC1 activation in normoxic cancer cells [33, 34]. Moreover, a previous study found MPC1 expression negatively associated with HIF1*α* expression in human RCC [35]. HIF1*α* is one of the key regulators in glycolysis as well as downstream of the mTOR pathway [36]. In combination with our current results, we speculate that MPC2 may inhibit glycolysis via the mTOR/HIF1*α* axis, which would be further confirmed in the future study.

In addition to the proliferation inhibiting role of MPC2, a study also investigated MPC1 losing promoted migration and invasion in RCC [35]. In our study, we also found that MPC2 expression is negatively associated with the incidence of lymph node invasion and liver metastasis. However, our study focused on the role of MPC2 in CRC growth. In the future, we would further explore whether MPC2 inhibits migration and invasion thus affecting liver metastasis in CRC using mouse models.

In summary, our results demonstrated decreased expression of MPC2 in CRC and its correlation with a poor prognosis of CRC patients. In addition, MPC2 inhibited CRC growth *in vivo* and *in vitro*. Moreover, MPC2 reduced glycolysis by inhibiting the mTOR pathway. Our study suggested a potential role of MPC2 in CRC diagnosis and a promising therapeutic target. The further underlying molecular mechanism remains to be determined and merits further study.

## 5. Conclusions

In conclusion, we found downregulated MPC2 expression in CRC tissues, as well as a negative association of the expression with distant metastasis, patients' survival, lymph node invasion, T stage, and tumor size. Thus, we also found that downregulated MPC2 promoted CRC growth by inducing glycolysis via the mTOR pathway. Our study suggested a potential role of MPC2 in CRC diagnosis and a promising therapeutic target.

## Figures and Tables

**Table 1:** Correlation of MPC2 expression with CRC patients' clinicopathological characteristics.

	MPC2		*p* value
	Low (*n* = 196)	High (*n* = 196)	
*Age (year)*	58.82 ± 8.45	59.74 ± 7.92	1.1766
*Sex*			
Male	110	122	0.2175
Female	86	74	
*Tumor size (cm)*	6.52 ± 2.08	4.48 ± 1.53	<0.0001
*T stage*			
T1	3	8	<0.01
T2	10	26	
T3	49	45	
T4	134	117	
*Lymph node invasion*			
Absent	82	129	<0.0001
Present	114	67	
*Distant metastasis*			
Absent	145	166	<0.01
Present	51	30	

## Data Availability

The data supporting the above findings can be accessed under the approval of the corresponding authors.

## Supplementary Materials

**S**upplementary Materials Supplementary Figure S1: A. The mRNA expression of MPC2 in several CRC cell lines performed by qRT-PCR. B. The overexpression efficiency in HT29 and SW620 cells showed using qRT-PCR. C. The viability of HT29 (left) and SW480 cells (right) transfected with MPC2-OE or vector detected with CCK-8 assay. C. The mRNA expression of MPC1 in HCT116 and RKO cells with MPC2 knockdown by shRNA measured by qRT-PCR (relative to shNC group). D. The mRNA expression of MPC1 in SW620 and HT29 cells with MPC2 overexpression measured by qRT-PCR (relative to vector group). E. The protein expression of MPC1 in HCT116 and RKO cells with MPC2 knockdown by shRNA measured by western blotting. G. The viability of SW480 cells (right) and HT29 cells (left) through the transfection by MPC2-OE or vector detected with CCK-8 assay. Supplementary Figure S2: MPC2 knockdown promoted glycolysis in CRC cells. A. Glycolytic flux measured by ECAR in HCT116 and RKO cells with MPC2 knockdown with/or MPC2 overexpression. B. Maximal respiratory capacity measured by OCR in HCT116 and RKO cells with MPC2 knockdown with/or MPC2 overexpression. Supplementary Figure S3: MPC2 overexpression inhibited glycolysis in CRC cells. A. Lactate production of CRC cells transfected with vector or MPC2-OE (relative to vector). B. Glucose consumption of CRC cells transfected with vector or MPC2-OE (relative to vector). C. Relative mRNA levels of glycolysis-related genes in HT29 cells (right) and SW620 cells (left) through the transfection via vector or MPC2-OE (relative to shNC). D. Glycolytic function of SW620 (left) and HT29 cells (right) under the indicated treatments showed by extracellular acidification rate (ECAR). E. Mitochondrial stress test glycolytic function of SW620 (left) and HT29 cells (right) in MPC2 knockdown and/or overexpression under the indicated treatments as measured by oxygen consumption rate (OCR). Supplementary Figure S4: MPC2 reduced p-mTOR level in CRC cells. A. Western blot analysis of phospho-mTOR (p-mTOR) and mTOR in SW620 and RKO cells with vector and MPC2 overexpression transfection and/or MHY1485 treatment (10 *μ*M, 4 h). B. Glycolytic flux measured by ECAR in HCT116 and RKO cells with MPC2 knockdown with/or rapamycin. B. Maximal respiratory capacity measured by OCR in HCT116 and RKO cells with MPC2 knockdown with/or rapamycin treatment (50 nmol/L). Supplementary Table S1: the number and percentage of IHC score in 392 cases of normal tissues and matched CRC tissues. Supplementary Table S2: sequences of shRNA and primer.
