# Supplementary material for: Decreased Expression of MPC2 Contributes to Aerobic Glycolysis and Colorectal Cancer Proliferation by Activating mTOR Pathway
Source: J Immunol Res. 2021 Mar 15;2021:6618837. doi: 10.1155/2021/6618837 (PMC7984920; doi:10.1155/2021/6618837)
Supplement: Supplementary Materials — Supplementary Figure S1: A. The mRNA expression of MPC2 in several CRC cell lines performed by qRT-PCR. B. The overexpression efficiency in HT29 and SW620 cells showed using qRT-PCR. C. The viability of HT29 (left) and SW480 cells (right) transfected with MPC2-OE or vector detected with CCK-8 assay. C. The mRNA expression of MPC1 in HCT116 and RKO cells with MPC2 knockdown by shRNA measured by qRT-PCR (relative to shNC group). D. The mRNA expression of MPC1 in SW620 and HT29 cells with MPC2 overexpression measured by qRT-PCR (relative to vector group). E. The protein expression of MPC1 in HCT116 and RKO cells with MPC2 knockdown by shRNA measured by western blotting. G. The viability of SW480 cells (right) and HT29 cells (left) through the transfection by MPC2-OE or vector detected with CCK-8 assay. Supplementary Figure S2: MPC2 knockdown promoted glycolysis in CRC cells. A. Glycolytic flux measured by ECAR in HCT116 and RKO cells with MPC2 knockdown with/or MPC2 overexpression. B. Maximal respiratory capacity measured by OCR in HCT116 and RKO cells with MPC2 knockdown with/or MPC2 overexpression. Supplementary Figure S3: MPC2 overexpression inhibited glycolysis in CRC cells. A. Lactate production of CRC cells transfected with vector or MPC2-OE (relative to vector). B. Glucose consumption of CRC cells transfected with vector or MPC2-OE (relative to vector). C. Relative mRNA levels of glycolysis-related genes in HT29 cells (right) and SW620 cells (left) through the transfection via vector or MPC2-OE (relative to shNC). D. Glycolytic function of SW620 (left) and HT29 cells (right) under the indicated treatments showed by extracellular acidification rate (ECAR). E. Mitochondrial stress test glycolytic function of SW620 (left) and HT29 cells (right) in MPC2 knockdown and/or overexpression under the indicated treatments as measured by oxygen consumption rate (OCR). Supplementary Figure S4: MPC2 reduced p-mTOR level in CRC cells. A. Western blot analysis of phospho-mTOR ( [file 6618837.f1.docx]

Supplementary Material


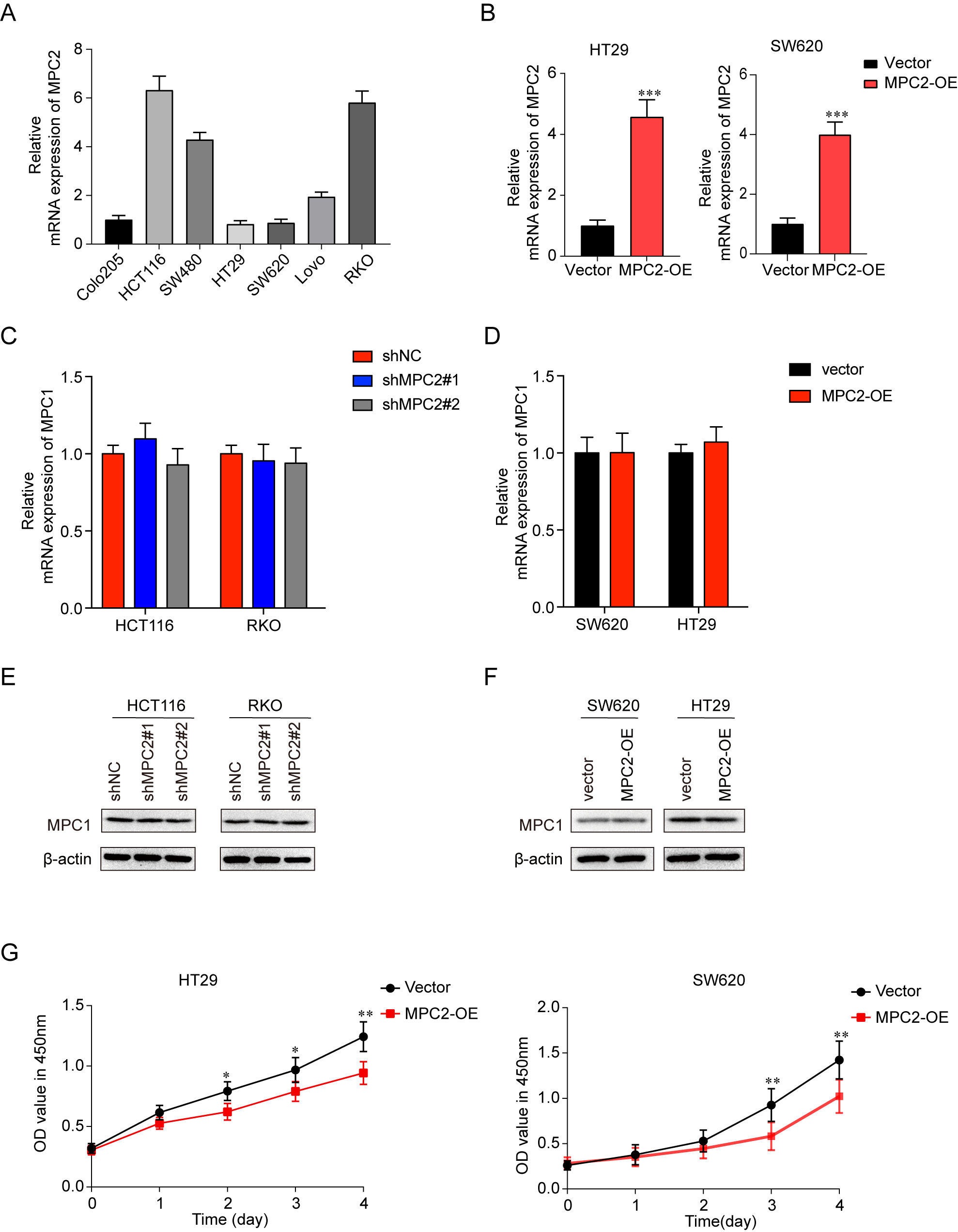


**Supplementary Figure S1.** A. The mRNA expression of MPC2 in seven CRC cell lines performed by qRT-PCR. B. The overexpression efficiency in HT29 and SW620 cells showed by qRT-PCR. C. The viability of HT29 (left) and SW480 cells (right) transfected with MPC2-OE or vector detected with CCK-8 assay. **C.** The mRNA expression of MPC1 in HCT116 and RKO cells with MPC2 knockdown by shRNA measured by qRT-PCR (relative to shNC group). D. The mRNA expression of MPC1 in SW620and HT29 cells with MPC2 overexpression measured by qRT-PCR (relative to vector group). **E.** The protein expression of MPC1 in HCT116 and RKO cells with MPC2 knockdown by shRNA measured by western blotting. **G.** The viability of HT29 (left) and SW480 cells (right) transfected with MPC2-OE or vector detected with CCK-8 assay.

**
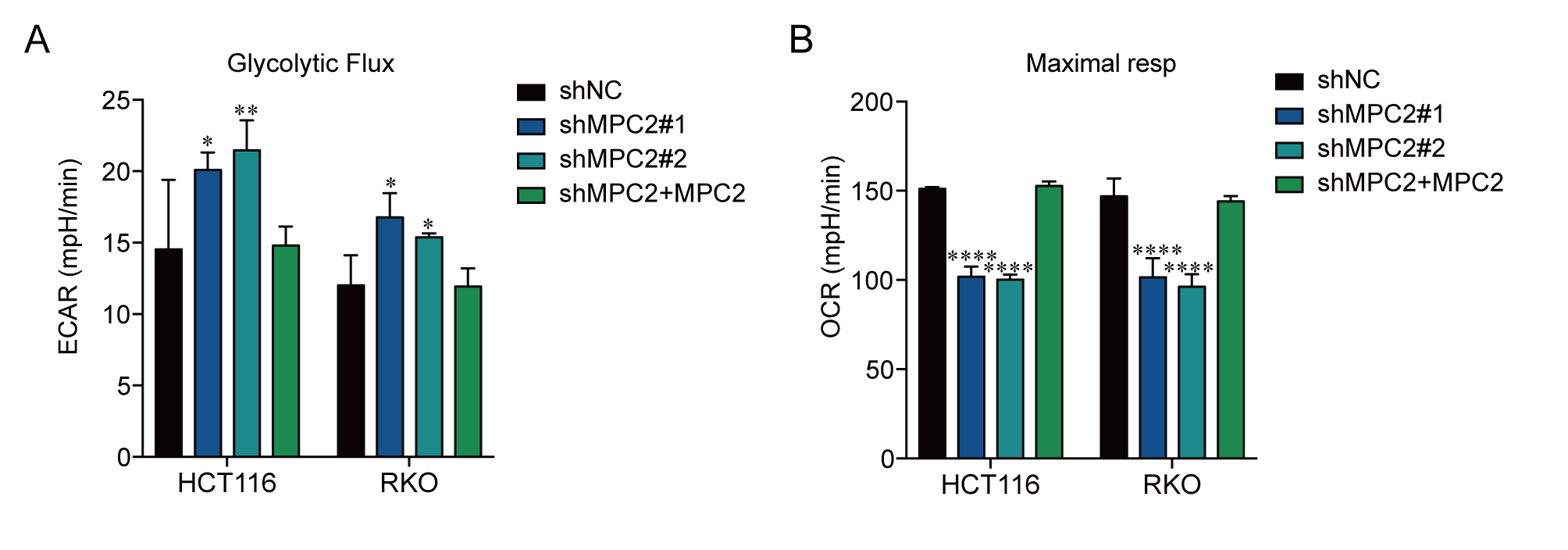
Supplementary Figure S2.** **The effect of MPC2 knockdown on glycolysis in CRC.A.** Glycolytic flux measured by ECAR in HCT116 and RKO cells with MPC2 knockdown with/or MPC2 overexpression. **B.** Maximal respiratory capacity measured by OCR in HCT116 and RKO cells with MPC2 knockdown with/or MPC2 overexpression.


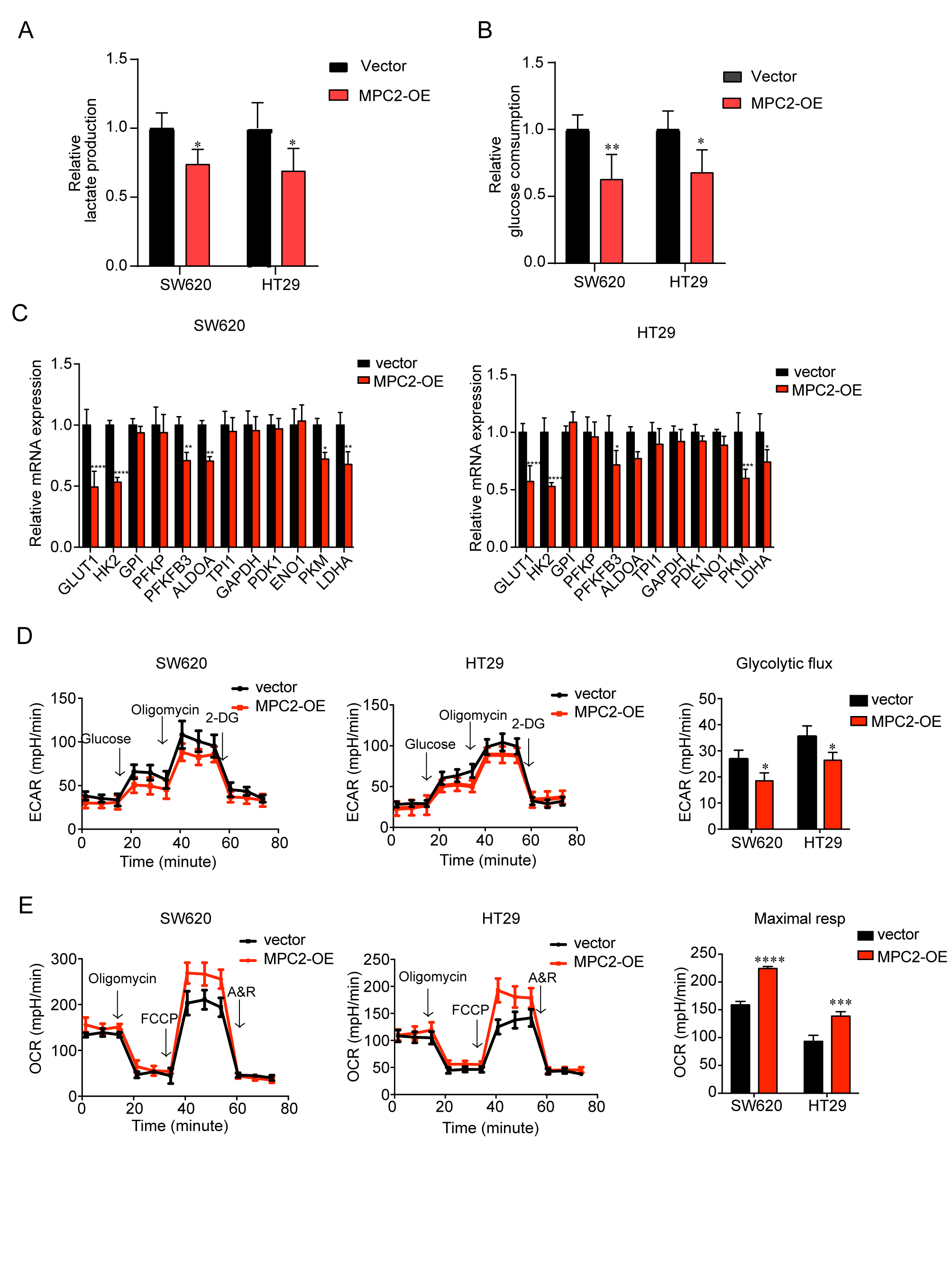


**Supplementary Figure S3.The effect of MPC2 overexpression on glycolysis in CRC. A.** Lactate production of CRC cells transfected with vector or MPC2-OE (relative to vector). **B.** Glucose consumption of CRC cells transfected with vector or MPC2-OE (relative to vector). **C.** Relative mRNA levels of glycolysis-related genes of SW620 (left) and HT29 cells (right) transfected with vector or MPC2-OE (relative to shNC). **D.** Glycolytic function of SW620 (left) and HT29 cells (right) under the indicated treatments as measured by extracellular acidification rate (ECAR). **E.** Mitochondrial stress test Glycolytic function of SW620 (left) and HT29 cells (right) in MPC2 knockdown and/or overexpression under the indicated treatments as measured by oxygen consumption rate (OCR).


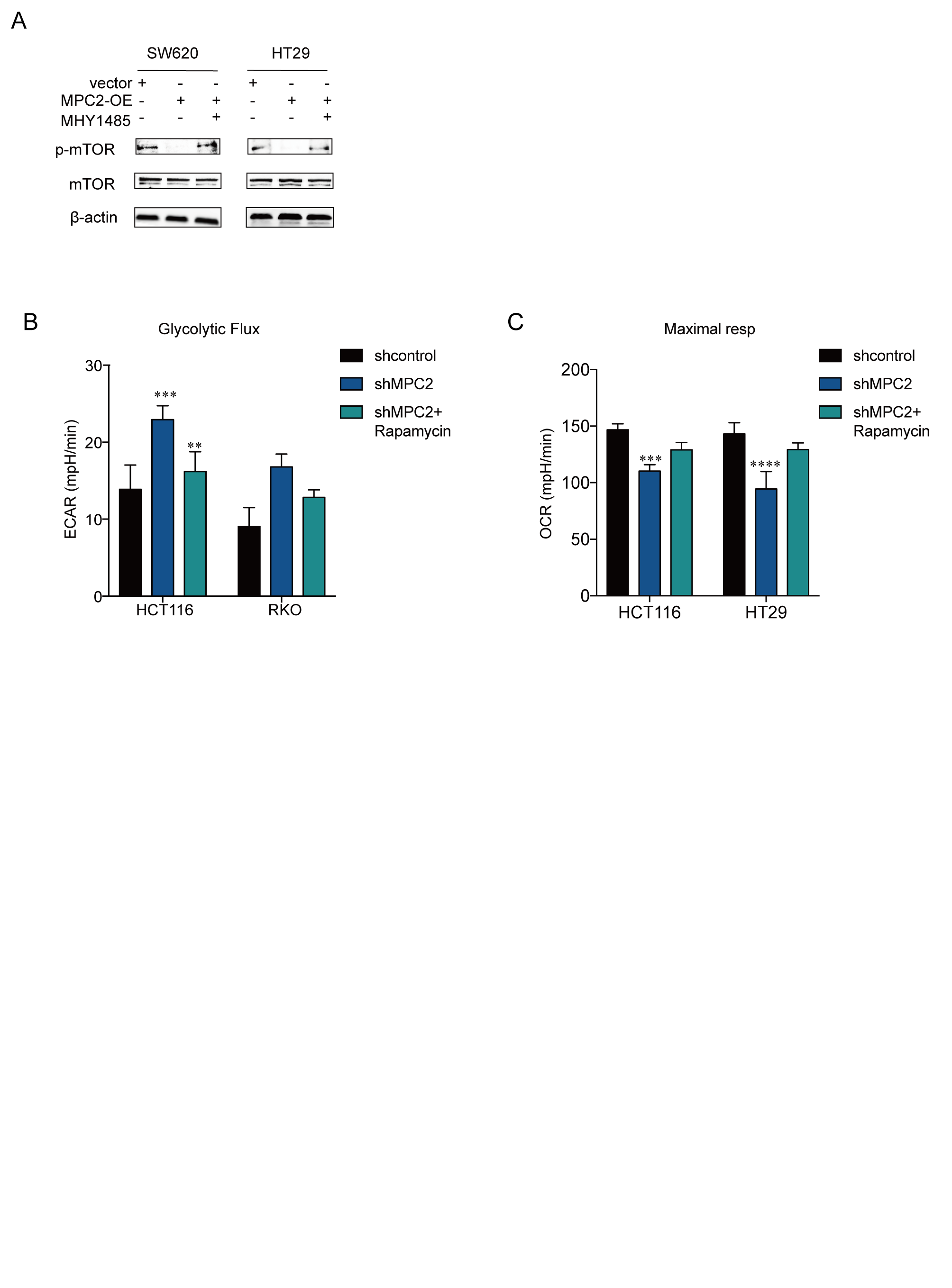


**Supplementary Figure S4.** **MPC2 reduced p-mTOR level in CRC cells. A.** Western blot analysis of phospho-mTOR (p-mTOR) and mTOR in SW620 and RKO cells with vector and MPC2 overexpression transfection and/or MHY1485 treatment (10uM,4h). **B.** Glycolytic flux measured by ECAR in HCT116 and RKO cells with MPC2 knockdown with/or rapamycin. **C.** Maximal respiratory capacity measured by OCR in HCT116 and RKO cells with MPC2 knockdown with/or rapamycin treatment (50 nmol/L).

**Supplementary Table S1. The number and precent of IHC score in 392 cases of normal tissues and matched CRC tissues.**

|  | MPC2 score | | | |  |
| --- | --- | --- | --- | --- | --- |
| Tissue | 1 | 2 | 3 | 4 | *P* |
| Normal | 74(19%) | 122(31%) | 110(28%) | 86(22%) |  |
| CRC | 172(44%) | 24(6%) | 145(37%) | 51(13%) | *0.0289* |

**Supplementary Table S2. Sequence of shRNA and primer**

|  | **sequence (5'--->3')** | |
| --- | --- | --- |
| MPC2 shNC | TGTTCTCCGAACGTGTCACGTTTCAAGAGAACGTGACACGTTCGGAGGATTTTTTC | |
|  | TCGAGAAAAAATTCTCCGAACGTGTCACGTTCTCTTGAAACGTGACACGTTCGGAGAACA | |
| MPC2 sh1 | TGACTATGTCCGAAGGATTTCAAGAGAATCCTTGCTTCGGACATAGTCTTTTTTC | |
|  | TCGAGAAAAAAGACTATGTCCGAAGCAAGGATTCTCTTGAAATCCTTGCTTCGGACATAGTCA | |
| MPC2 sh2 | TGCTCCTCGATAAAGTGGAGCTTTCAAGAGAAGCTCCACTTTATCGAGGAGCTTTTTTC | |
|  | TCGAGAAAAAAGCTCCTCGATAAAGTGGAGCTTCTCTTGAAAGCTCCACTTTATCGAGGAGCA | |
| 18S-F | TGCGAGTACTCAACACCAACA | |
| 18S-R | GCATATCTTCGGCCCACA |  |
| MPC2-F | TACCACCGGCTCCTCGATAAA | |
| MPC2-R | TATCAGCCAATCCAGCACACA | |
| GLUT1-F | ATTGGCTCCGGTATCGTCAAC | |
| GLUT1-R | GCTCAGATAGGACATCCAGGGTA |  |
| HK2-F | TGATCGCCTGCTTATTCACGG |  |
| HK2-R | AACCGCCTAGAAATCTCCAGA |  |
| GPI-F | CAAGGACCGCTTCAACCACTT | |
| GPI-R | CCAGGATGGGTGTGTTTGACC | |
| PFKP-F | CGCCTACCTCAACGTGGTG | |
| PFKP-R | ACCTCCAGAACGAAGGTCCTC | |
| PFKFB3-F | ATTGCGGTTTTCGATGCCAC |  |
| PFKFB3-R | GCCACAACTGTAGGGTCGT |  |
| ALDOA-F | ATGCCCTACCAATATCCAGCA | |
| ALDOA-R | GCTCCCAGTGGACTCATCTG | |
| TPI1-F | CTCATCGGCACTCTGAACG | |
| TPI1-R | GCGAAGTCGATATAGGCAGTAGG | |
| GAPDH-F | GGAGCGAGATCCCTCCAAAAT | |
| GAPDH-R | GGCTGTTGTCATACTTCTCATGG | |
| PDK1-F | CTGTGATACGGATCAGAAACCG | |
| PDK-R | TCCACCAAACAATAAAGAGTGCT | |
| ENO1-F | AAAGCTGGTGCCGTTGAGAA | |
| ENO1-R | GGTTGTGGTAAACCTCTGCTC | |
| PKM-F | TCGCATGCAGCACCTGATT |  |
| PKM-R | CCTCGAATAGCTGCAAGTGGTA |  |
| LDHA-F | GCTCCCCAGAACAAGATTACAG |  |
| LDHA-R | TCGCCCTTGAGTTTGTCTTC |  |
